# Theoretical Knowledge, Self-Assessed Competence, and Information-Seeking Behaviors in Evidence-Based Medicine Among Polish Medical Students: A Cross-Sectional Study

**DOI:** 10.7759/cureus.116364

**Published:** 2026-09-16

**Authors:** Bartosz Kedziora, Ewelina Glowacka, Paulina Kowalska, Wiktor Kaczmarek, Joanna Kedziora, Karolina Osowiecka, Ewa Orlewska

**Affiliations:** 1 College of Medicine, Jan Kochanowski University, Kielce, POL; 2 Department of Anesthesiology and Intensive Care, Provincial Combined Hospital, Kielce, POL; 3 Department of Cardiology, Municipal Hospital, Olsztyn, POL; 4 Second Department of Pediatrics, Provincial Combined Hospital, Kielce, POL; 5 School of Public Health, University of Warmia and Mazury in Olsztyn, Olsztyn, POL

**Keywords:** behavior, ebm competence, evidence-based medicine, medical education, medical students, self-assessment

## Abstract

Background

The effectiveness of Evidence-Based Medicine (EBM) education at the undergraduate level remains uncertain, particularly regarding its impact on objective knowledge, self-perceived competence, and information-seeking behavior. This study aimed to provide a comprehensive assessment of EBM preparedness among medical students in Poland.

Methods

A cross-sectional survey was conducted among 329 medical students from multiple Polish universities. The questionnaire combined self-assessment of EBM knowledge and skills, a 10-item objective knowledge test, and evaluation of students’ preferred sources of medical information. Associations between EBM course participation and outcomes were analyzed using nonparametric tests and correlation analyses.

Results

The mean knowledge test score was 4.75 ± 2.33 (median 5/10), indicating moderate overall knowledge with substantial variability. Participation in EBM courses was associated with higher test scores (5.12 vs. 4.21; p = 0.001) and fewer “I do not know” responses (p = 0.002). A strong correlation was observed between self-assessed theoretical knowledge and objective performance (rho = 0.67; p < 0.001). Students with EBM training more frequently used scientific articles (p = 0.009), clinical guidelines (p < 0.001), and scientific databases (88.7% vs. 50.4%; p < 0.001), with database use positively correlated with test scores (rho = 0.249; p < 0.001). Authorship or co-authorship of scientific publications was consistently associated with higher knowledge scores and self-assessed competencies (p < 0.001).

Conclusions

Formal EBM education is associated with measurable improvements in knowledge and promotes the use of evidence-oriented information sources. However, overall knowledge remains moderate, suggesting that current teaching approaches are insufficient. Active engagement in scientific research appears to reinforce EBM competencies and should be considered an integral component of EBM education. Effective EBM teaching may therefore require not only structured coursework but also systematic encouragement of student involvement in research and scientific writing.

## Introduction

Evidence-Based Medicine (EBM) has become a fundamental component of contemporary clinical practice. It is not limited to the passive use of published data but requires the ability to search for evidence efficiently, critically appraise its methodological quality, and apply it in the context of an individual patient’s needs and values ​[1-3].

Teaching EBM at the undergraduate level presents both opportunities and challenges. On the one hand, early exposure to the principles of critical appraisal and basic medical statistics may help students develop habits that persist throughout their professional careers. On the other hand, numerous studies suggest that the level of EBM knowledge and skills among medical students varies considerably across institutions and countries. The available literature does not provide a uniform picture ​[2-6].

In Hungary, medical students demonstrated satisfactory knowledge of EBM, and their understanding of specific concepts improved after completing a dedicated course [2]. In contrast, studies conducted in Sudan reported substantial gaps in students’ familiarity with EBM terminology and skills, with limited impact of formal classes on attitudes toward EBM [3,7]. Similarly, low levels of EBM knowledge were observed among Iranian and Syrian students, particularly in contexts where EBM was not formally integrated into the curriculum [4,8]. Data from the United States point to a measurable discrepancy between expected and actual knowledge levels before entering residency training [9]. In the Netherlands and Norway, students assessed their own EBM knowledge as insufficient to average and relied predominantly on textbooks and supervisors’ opinions when addressing clinical questions [10,11]. Discrepancies between self-assessed and objectively measured competence were also reported in other research [12,13]. Similar gaps between reported curricular exposure and practical competencies were reported among students in Saudi Arabia and Oman [5,6]. Similarly, research in Egypt showed that formal EBM training did not always translate into consistent knowledge, practices, or attitudes among physicians [14]. Comparative analyses of different EBM knowledge assessment tools further indicate that measured student performance may vary depending on the instrument used [15].

These findings suggest that the effectiveness of EBM education may depend on curricular structure, intensity of training, and broader educational culture. At the same time, most studies focus either on self-reported competence or on selected aspects of knowledge, rarely combining objective assessment, self-evaluation, and analysis of information-seeking behaviors within a single study design [12,15,16].

Despite the growing emphasis on EBM in medical education, comprehensive data regarding Polish medical students remain limited. Although many medical faculties in Poland formally include EBM-related subjects in their curricula, the extent to which these courses translate into measurable knowledge and practical information-seeking habits has not been systematically examined.

The present study was designed to assess both theoretical knowledge and self-perceived competencies in EBM among medical students in Poland. In addition to testing familiarity with core EBM concepts and basic statistical terminology, we evaluated students’ ability to recognize the hierarchy of scientific evidence and explored the sources of medical information they most frequently use. We hypothesized that participation in formal EBM courses would be associated with higher levels of objective knowledge, greater confidence in EBM-related skills, and more frequent use of evidence-oriented information sources.

By combining objective testing, self-assessment, and analysis of knowledge sources, this study aims to provide a comprehensive overview of EBM preparedness among Polish medical students and to identify areas that may require further educational attention.

## Materials and methods

Study design and setting

A cross-sectional survey study was conducted among Polish-speaking medical students between May 2020 and November 2020, and between June 2025 and August 2025. A total of 341 respondents participated in the study.

Ethical approval

The study protocol was approved by the Ethics Committee of the University of Warmia and Mazury in Olsztyn (approval no. 4/2023). Participation was voluntary and anonymous; no incentives were offered, and respondents gave their consent to participate in the study by starting to fill in the survey.

Participants

The study population consisted exclusively of medical students studying in the Polish language. Students from 21 Polish medical universities were invited to participate (the list of these institutions is provided in Appendix A). Participants were recruited using a mixed recruitment strategy combining in-person and electronic survey distribution. At the University of Warmia and Mazury in Olsztyn, paper-based questionnaires were distributed in person to medical students from all academic years during the examination session. For other Polish medical universities, the questionnaire was distributed in electronic form. Formal requests were submitted to university authorities asking for permission to disseminate the survey among medical students via official institutional channels. Three universities granted formal approval and actively distributed the survey. Several institutions did not provide an official response but nonetheless allowed the questionnaire to be shared informally. In contrast, some universities declined participation or did not disseminate the survey at all. Four universities did not yield any completed questionnaires. In addition, to increase outreach and participation, the authors sought support from the International Federation of Medical Students' Associations - Poland, which assisted in circulating the electronic survey among medical students nationwide. Additionally, respondents who reported studying medicine in Poland but were not enrolled in the listed universities were categorized as “Other.” No additional exclusion criteria were applied. A visual representation of the student selection process is presented in Figure 1.

**Figure 1:**
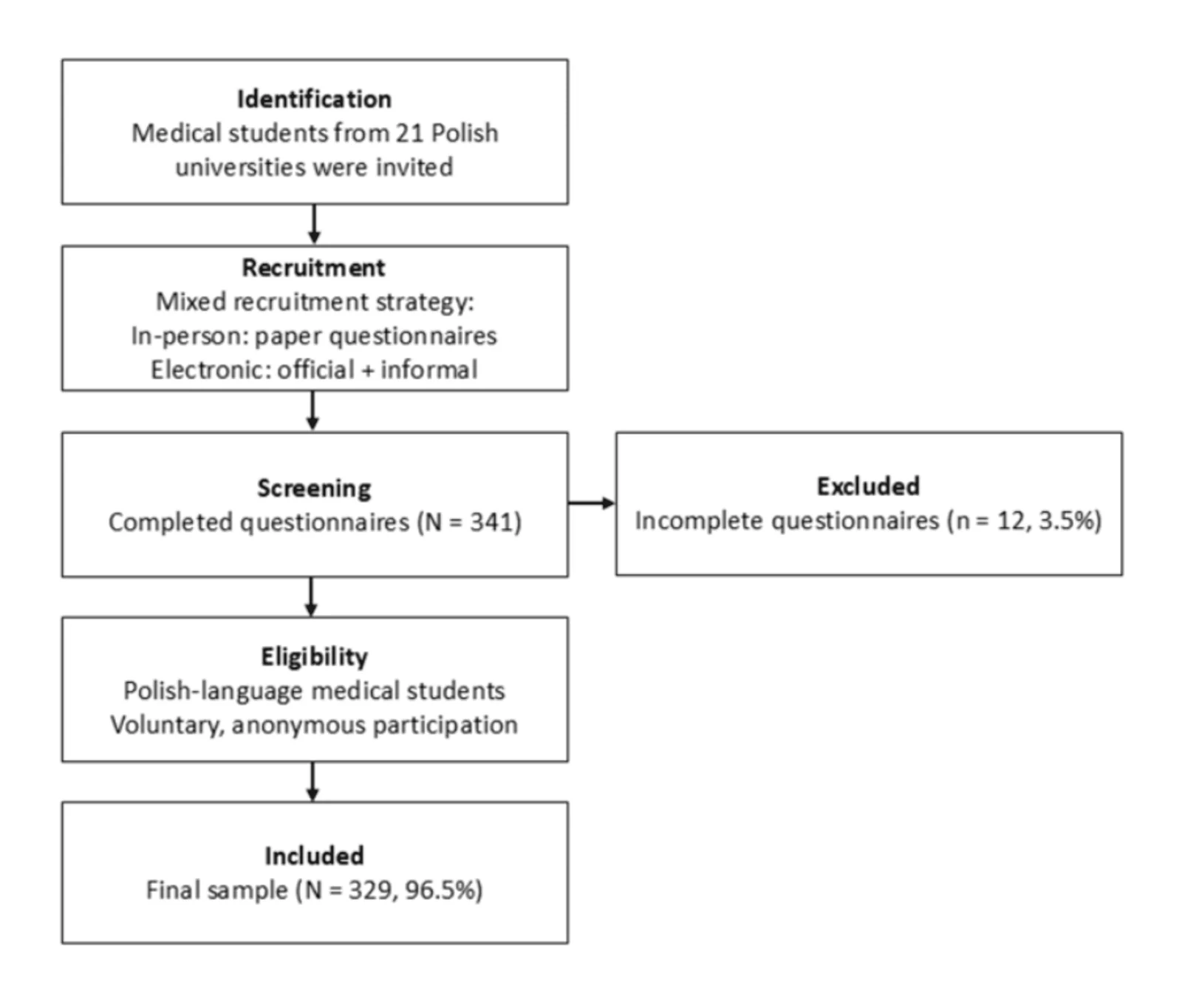
Flow diagram of study participant selection process

Questionnaires

The questionnaire was originally developed in accordance with general principles of test development to assess medical students’ knowledge and competencies in EBM. Particular attention was paid to minimizing potential sources of measurement bias, including leading questions, overly complex wording, and ambiguous response options. Prior to full deployment, the questionnaire underwent pilot testing to assess clarity and comprehensibility. The complete questionnaire is provided in Appendix B. The questionnaire consisted of three conceptual sections:

General Participant Information

This section collected demographic and educational data, including university affiliation, year of study, possession of a prior bachelor’s, master’s, or engineering degree, presence of family members working in healthcare or scientific professions, and authorship or co-authorship of scientific publications. Participants were also asked about their primary sources of scientific information, prior participation in EBM courses, and their awareness of EBM courses offered at their university.

Self-Assessment of EBM Knowledge and Skills

This section comprised 28 dichotomous (“yes/no”) items assessing familiarity with EBM concepts (14 items) and statistical terminology (14 items). In addition, participants rated their perceived competence on a five-point Likert scale (1 = poor, 5 = strong) in two domains: (Skill 1) critical appraisal of scientific literature and (Skill 2) identification of clinical questions lacking sufficient high-quality evidence.

Objective Assessment of EBM Knowledge

This section consisted of a knowledge-based quiz including 10 single-choice, closed-ended questions. Each question offered four response options: one correct answer, two incorrect answers, and one “I do not know” option. For the objective knowledge test, each correct answer was awarded 1 point, while incorrect and "I do not know” responses were awarded 0 points. The total score ranged from 0 to 10 points. The final task required participants to rank six types of research designs according to their perceived credibility, from 1 (“least credible”) to 6 (“most credible”). The study designs included: case report, non-randomized controlled trial, observational controlled trial, single randomized controlled trial, systematic review of observational studies, and meta-analysis of randomized controlled trials. Items in Sections 2 and 3 were presented in a non-fixed order and grouped thematically to reduce potential order effects.

Response rate

Of all questionnaires distributed in 2020 and 2025, a total of 341 were completed. A total of 12 questionnaires (3.5%) were excluded due to incomplete data.

Pilot testing and reliability assessment

After the first 20 students completed the questionnaire, structured interviews were conducted to verify that all items were clearly understood as intended. Test-retest reliability was assessed by asking the same respondents to complete the selected items again after two weeks. Questions 1-12 (demographic and background items) and Question 25 (a ranking item) were not included in the reliability assessment. For all assessed questions, the significance test indicated that Cohen's kappa coefficient was significantly different from 0, suggesting that the measurements were consistent (repeatable). Very good repeatability was observed for 19 questions (13.1, 13.2, 13.11, 13.16, 13.17, 13.18, 13.19, 13.22, 13.23, 13.24, 13.26, 13.27, 13.28, 14.2, 16, 17, 18, 21, and 24), good repeatability for 18 questions (13.3, 13.4, 13.5, 13.6, 13.8, 13.9, 13.10, 13.12, 13.13, 13.14, 13.15, 13.25, 14.1, 15, 19, 20, 22, and 23), and moderate repeatability for three questions (13.7, 13.20, and 13.21). Detailed results of the reliability analysis are provided in Appendix C.

Statistical analysis

The data were presented as means and standard deviations for continuous variables and as frequencies and percentages for categorical variables. The normality of data distribution was tested using the Shapiro-Wilk test. Comparisons between two independent groups (e.g., students who participated in EBM courses vs. those who did not; students with and without publication experience; students with and without prior university degrees) were performed using the Mann-Whitney U test. Effect sizes for the Mann-Whitney test were calculated using the rank-biserial correlation coefficient (r), interpreted according to conventional thresholds (small ≈ 0.1, moderate ≈ 0.3, large ≥ 0.5). Comparisons across more than two independent groups (e.g., year of study) were conducted using the Kruskal-Wallis test. Associations between ordinal or non-normally distributed continuous variables (e.g., test scores, number of “I don’t know” responses, number of online sources used) were assessed using Spearman’s rank correlation coefficient (rho). Associations between categorical variables (e.g., use of specific information sources, participation in EBM courses, authorship of scientific publications) were analyzed using the chi-square (χ^2^) test of independence. When expected cell counts were low, Fisher’s exact test was applied. For paired nominal comparisons within the same subgroup (e.g., comparison of use of medical portals vs. databases among younger students), McNemar’s test was used. Effect sizes for categorical associations were estimated using Cramér’s V. For the hierarchy of evidence task, overall performance was assessed both categorically (correct vs. incorrect ranking for each study type) and quantitatively using a total classification error score, defined as the sum of absolute differences between the rank assigned by the participant and the predefined correct rank. Lower values indicated greater agreement with the reference hierarchy. All statistical tests were two-tailed. A p-value < 0.05 was considered statistically significant. All statistical analyses and figures were generated in the Python environment (Python Software Foundation, Fredericksburg, VA, USA) using Jupyter Notebook (version 7.3.2) with the SciPy (version 1.16.3), NumPy (version 2.2.0), pandas (version 2.2.3), and matplotlib (version 3.10.0) libraries.

## Results

Characteristics of survey participants

The final study sample consisted of 329 medical students (years I-VI) from different universities across Poland. Detailed participant characteristics are presented in Appendix A. Twenty-three participants (7.0%) reported having obtained a prior higher education degree. Authorship or co-authorship of a published scientific paper was declared by 23 respondents (7.0%). Ninety-two participants (28.0%) reported having a physician in their close family, and 61 (18.5%) reported having a family member working as a scientist. One hundred and ninety-four respondents (59.0%) declared having completed an EBM course during their studies. Two hundred and fourteen students (65.0%) confirmed that EBM courses were included in their faculty curriculum. However, 97 students (29.5%) reported being unaware of such courses.

Self-reported skills in EBM

Self-assessment scores differed significantly between students who had participated in EBM-related courses and those who had not, favoring the EBM-trained group (p < 0.001). The relationship between self-assessed theoretical knowledge and performance in the objective knowledge test was subsequently examined, and a strong correlation was observed (rho = 0.67; p < 0.001). Additional analyses were conducted to examine associations between self-assessed theoretical knowledge and selected participant characteristics. A statistically significant positive correlation was found between year of study and self-assessment score (rho = 0.435; p < 0.001). No statistically significant differences were observed between students with and without a prior university degree (p = 0.169). Similarly, no statistically significant differences in self-assessment scores were observed between students with and without a close family member working as a physician (p = 0.213), a scientist (p = 0.301), or either a physician or a scientist (p = 0.262). Students who reported authorship or co-authorship of a published scientific article demonstrated significantly higher self-assessment scores than those without such experience (p < 0.001). Results of the self-assessment of EBM knowledge are presented in Table 1.

**Table 1:** Self-assessment of theoretical knowledge among respondents (N = 329)

Term	Yes	No
Original article	237 (72.0%)	92 (28.0%)
Review article	239 (72.6%)	90 (27.4%)
Case report	314 (95.4%)	15 (4.6%)
Cohort study	132 (40.1%)	197 (59.9%)
Prospective study	152 (46.2%)	177 (53.8%)
Retrospective study	193 (58.7%)	136 (41.3%)
Cross-sectional study	187 (56.8%)	142 (43.2%)
Case-control study	192 (58.4%)	137 (41.6%)
Phase I clinical trial	184 (55.9%)	145 (44.1%)
Phase II clinical trial	183 (55.6%)	146 (44.4%)
Phase III clinical trial	183 (55.6%)	146 (44.4%)
Medical experiment	274 (83.3%)	55 (16.7%)
Meta-analysis	202 (61.4%)	127 (38.6%)
Systematic review	151 (45.9%)	178 (54.1%)
p-value	177 (53.8%)	152 (46.2%)
Confidence interval	137 (41.6%)	192 (58.4%)
Median	316 (96.0%)	13 (4.0%)
Mean	323 (98.2%)	6 (1.8%)
Mode	230 (69.9%)	99 (30.1%)
Standard deviation	283 (86.0%)	46 (14.0%)
Type I error	93 (28.3%)	236 (71.7%)
Type II error	92 (28.0%)	237 (72.0%)
Odds ratio	90 (27.4%)	239 (72.6%)
Specificity	214 (65.0%)	115 (35.0%)
Sensitivity	232 (70.5%)	97 (29.5%)
Relative risk	122 (37.1%)	207 (62.9%)
Randomization	237 (72.0%)	92 (28.0%)
Evidence-based medicine	245 (74.5%)	84 (25.5%)

Self-assessment of practical skills

The most frequently reported rating for both skills was 3 (“average”), reported by 114 participants (34.6%) for Skill 1 (Figure 2) and 106 (32.2%) for Skill 2 (Figure 3). The highest rating (5) was reported by 31 participants: 17 (5.2%) for Skill 1 and 14 (4.3%) for Skill 2, whereas the lowest rating (1) was reported by 98 participants: 36 (10.9%) for Skill 1 and 62 (18.8%) for Skill 2.

**Figure 2:**
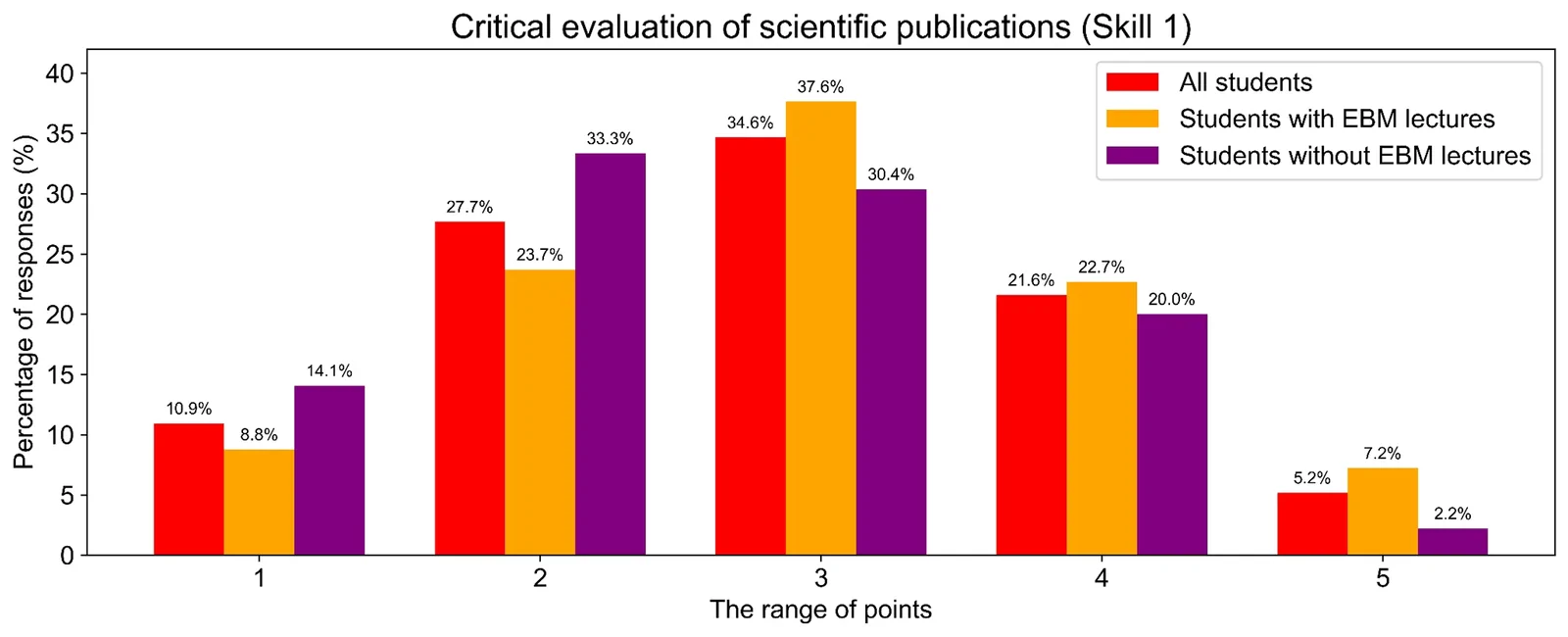
Self-assessment scores of practical skill: critical evaluation of scientific publications (Skill 1) EBM: Evidence-Based Medicine

**Figure 3:**
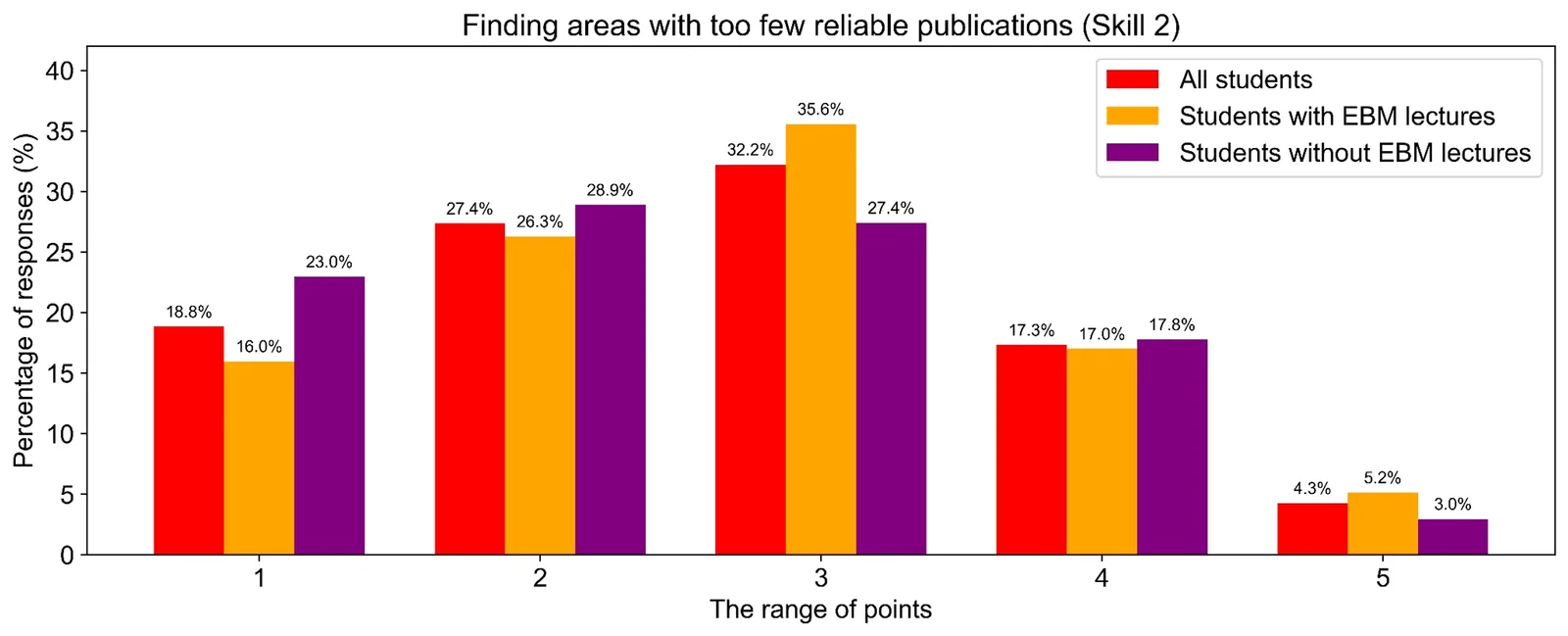
Self-assessment scores of practical skill: finding areas with too few reliable publications (Skill 2) EBM: Evidence-Based Medicine

Self-assessment scores were compared between students who had participated in academic EBM courses and those who had not. In the EBM group, the highest rating (5) for Skill 1 was reported more frequently than in the non-EBM group: 14 responses (7.2%) vs. three responses (2.2%), while the lowest rating (1) occurred more frequently in the non-EBM group: 19 responses (14.1%) vs. 17 responses (8.8%). For Skill 2, the highest rating (5) was reported 10 times in the EBM group (5.2%) and four times in the non-EBM group (3.0%), whereas the lowest rating (1) occurred in 31 cases in both groups, representing 16.0% of the EBM group and 23.0% of the non-EBM group. The distributions of self-assessment scores differed statistically significantly between groups for Skill 1 (p = 0.003), with higher scores observed in the EBM group; however, the effect size was small (r = 0.145). For Skill 2, the difference did not reach statistical significance (p = 0.053).

The relationship between self-assessed practical skills and performance on the objective knowledge test was examined. For Skill 1, a statistically significant moderate positive correlation with test scores was observed (rho ≈ 0.33, p < 0.001). For Skill 2, the correlation with test scores was statistically significant but weak (rho ≈ 0.17, p < 0.001).

Additional analyses examined associations between self-assessed practical skills and selected participant characteristics. A weak but statistically significant association was observed between year of study and Skill 1 self-assessment (rho = 0.15; p = 0.018), whereas no significant association was observed for Skill 2 (rho = -0.01; p = 0.440). No statistically significant differences in self-assessment of practical skills were observed between students with and without a prior university degree, for either Skill 1 (p = 0.108) or Skill 2 (p = 0.242). Finally, students who reported authorship or co-authorship of a scientific publication demonstrated significantly higher self-assessment scores for both Skill 1 (p = 0.0002) and Skill 2 (p = 0.003).

Results of the EBM knowledge test: knowledge of EBM-related terms

Scores in the theoretical knowledge test ranged from 0 to 10 points (Figure 4). The mean score was 4.75 ± 2.33, with a median of 5. The lower quartile (Q1) was 3, and the upper quartile (Q3) was 6. The highest proportion of correct responses was observed for the item assessing the definition of EBM (n = 269, 81.8%) (Table 2). The lowest proportion of correct responses was recorded for the item concerning relative risk in clinical trials (n = 68, 20.7%). The “I do not know” option was selected most frequently for the item addressing type I error (n = 196, 59.6%) and least frequently for the item concerning standard deviation (n = 35, 10.6%). The maximum test score (10 points) was achieved by eight participants (2.4%), whereas the minimum score (0 points) was observed in nine participants (2.7%). The most frequently obtained score was 4 points (n = 54, 16.4%). Very low scores (0-1 point) were uncommon and were observed in 26 participants (7.9%).

**Figure 4:**
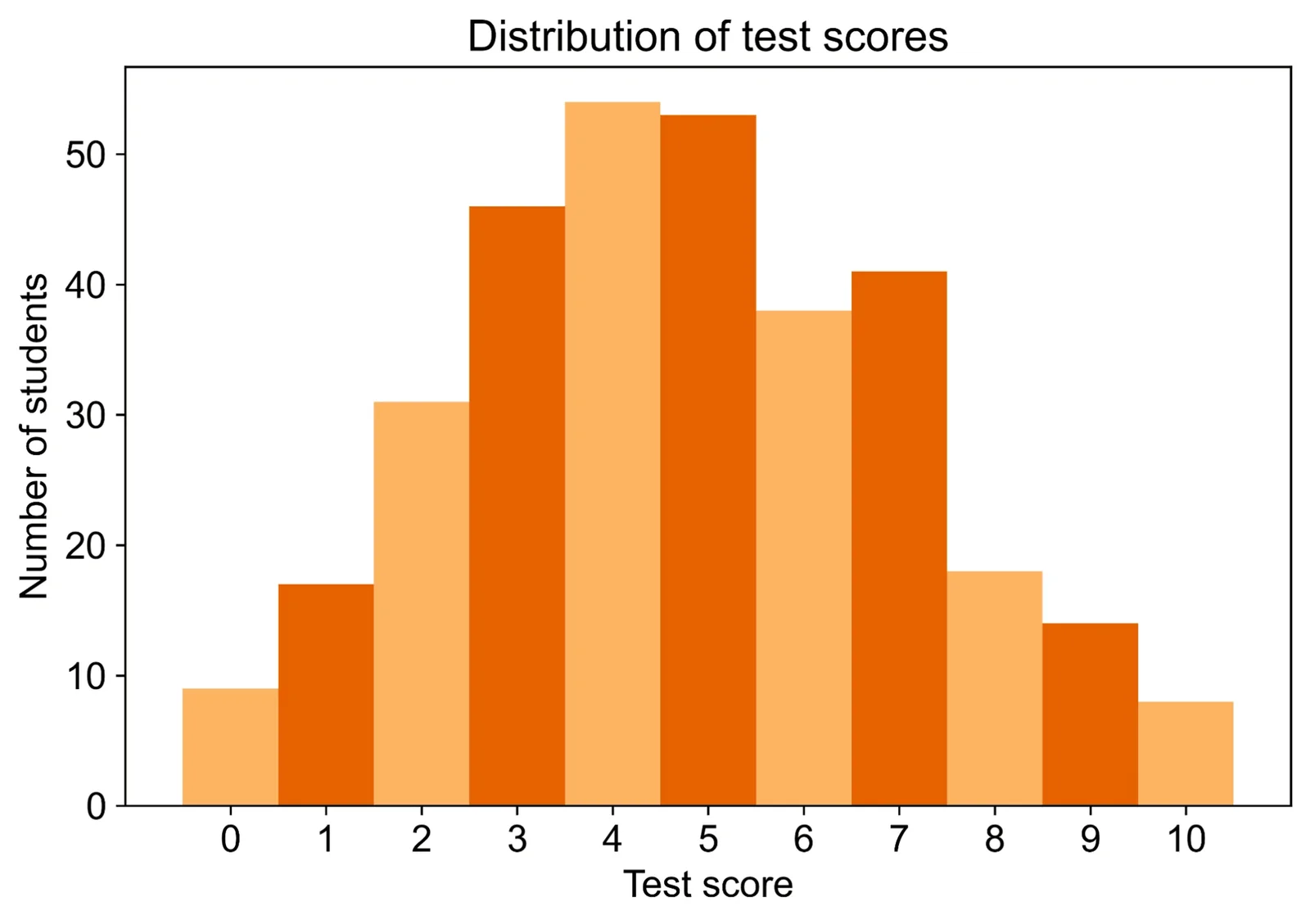
Distribution of theoretical knowledge test scores (0-10 points)

**Table 2:** Distribution of responses in the theoretical knowledge test

Term	Correct	I do not know	Incorrect
Cross-sectional study	101 (30.7%)	119 (36.2%)	109 (33.1%)
Case-control study	114 (34.7%)	111 (33.7%)	104 (31.6%)
Mode	216 (65.7%)	70 (21.3%)	43 (13.1%)
Standard deviation	191 (58.1%)	35 (10.6%)	103 (31.3%)
Type I error	83 (25.2%)	196 (59.6%)	50 (15.2%)
Confidence interval	154 (46.8%)	140 (42.6%)	35 (10.6%)
Phase II clinical trial	126 (38.3%)	134 (40.7%)	69 (21.0%)
Relative risk	68 (20.7%)	170 (51.7%)	91 (27.7%)
Randomization	241 (73.3%)	67 (20.4%)	21 (6.4%)
Evidence-based medicine (EBM)	269 (81.8%)	48 (14.6%)	12 (3.6%)

Five respondents (1.5%) selected the “I do not know” option for all test items, whereas 81 respondents (24.6%) did not select this option for any item. The median number of “I do not know” responses per participant was 3 (Q1 = 1; Q3 = 5) (Figure 5).

**Figure 5:**
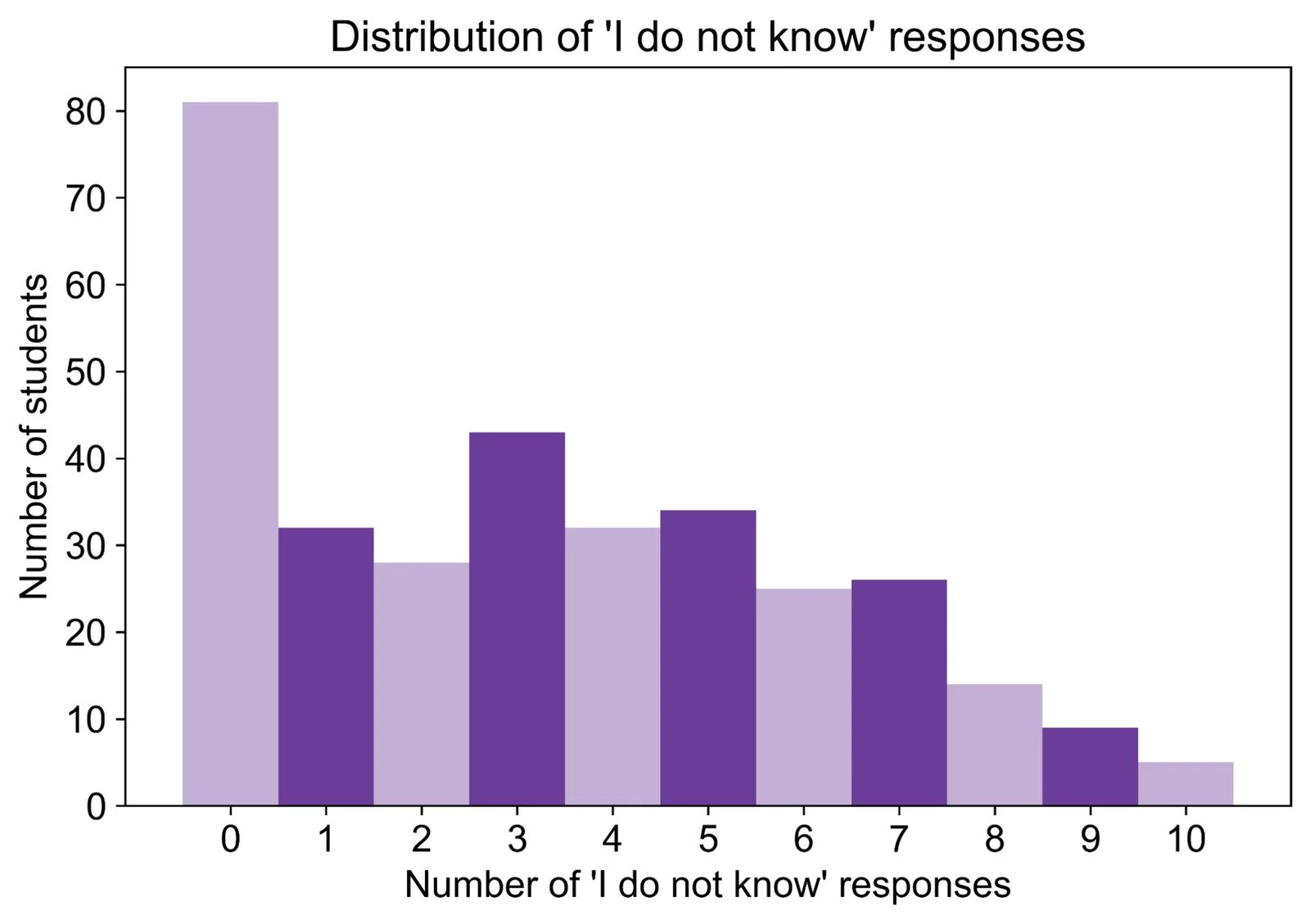
Distribution of the number of “I do not know” responses in the theoretical knowledge test

Associations between selected participant characteristics and performance in the theoretical knowledge test were examined, including year of study, participation in EBM courses, authorship of scientific publications, family background in medicine or science, and possession of a prior university degree. A statistically significant association was observed between year of study and test performance. Higher years of study were associated with higher test scores (rho = 0.27; p < 0.001) and a lower number of “I do not know” responses (rho = -0.21; p < 0.001). Between-group comparisons using the Kruskal-Wallis test confirmed significant differences between academic years for both test scores (p < 0.001) and the number of “I do not know” responses (p = 0.001). Students who reported participation in EBM courses achieved higher mean scores on the theoretical knowledge test (5.12 ± 2.17) compared with those who had not attended such courses (4.21 ± 2.45), and this difference was statistically significant (p = 0.001). Participants who had attended EBM courses also selected the “I do not know” response less frequently (p = 0.002).

Students who reported authorship or co-authorship of a scientific publication achieved significantly higher test scores than those without publication experience (p < 0.001) and selected the “I do not know” response less frequently (p < 0.001). Having a close family member who was a physician was associated with slightly higher test scores; however, this difference did not reach statistical significance (p = 0.095), nor did it significantly affect the number of “I do not know” responses (p = 0.26). No statistically significant associations were observed for having a scientist in the family. Students who had already obtained a prior university degree achieved slightly higher test scores, although this difference did not reach statistical significance. In contrast, graduates selected the “I do not know” response significantly less frequently than non-graduates (p = 0.013).

Results of the hierarchy test are presented in Table 3. The highest proportion of correct rankings was observed for the extreme categories of the evidence hierarchy. Correct ranks were assigned by 233 respondents (70.8%) for case reports and by 217 (66.0%) for meta-analyses of randomized controlled trials. The lowest proportion of correct rankings was observed for systematic reviews and single randomized controlled trials, for which correct ranks were assigned by 30 (9.1%) and 63 (19.2%) of respondents, respectively. Comparisons between students who had attended EBM courses and those who had not demonstrated higher accuracy of ranking in the EBM group for five of the six study types. No statistically significant difference between groups was observed for the systematic review category. Complete correct ranking of all six study types (6/6) was achieved by 13 students (6.7%) in the EBM group and by three students (2.2%) in the non-EBM group. This difference did not reach statistical significance (Fisher’s exact test, p = 0.072). In addition, total classification error was calculated as the sum of absolute differences between the rank assigned by a participant and the rank defined as correct. Students who had attended EBM courses demonstrated a significantly lower total classification error compared with those who had not (mean error: 5.88 vs 8.35, respectively; p < 0.001).

**Table 3:** Accuracy of study design ranking according to the hierarchy of evidence, overall and by participation in EBM course EBM: Evidence-Based Medicine

Correct order	Type of clinical trial	Total number of correct answers (N = 329)	Number of correct answers in group after EBM course (n = 194)	Number of correct answers in group without EBM lecture (n = 135)
1	Case report	233 (70.8%)	157 (80.9%)	76 (56.3%)
2	Single observational controlled study	103 (31.3%)	76 (39.2%)	27 (20.0%)
3	Systematic review of observational studies	30 (9.1%)	19 (9.8%)	11 (8.2%)
4	Nonrandomized controlled experimental study	109 (33.1%)	76 (39.2%)	33 (24.4%)
5	Single randomized controlled trial	63 (19.2%)	45 (23.2%)	18 (13.3%)
6	Meta-analysis of randomized controlled trials	217 (66.0%)	146 (75.3%)	71 (52.6%)

Students who reported authorship or co-authorship of a scientific publication demonstrated higher accuracy in assigning correct ranks for individual study types. Statistically significant differences between students with and without publication experience were observed for four of the six study categories; no significant differences were found for systematic reviews or single randomized controlled trials. Complete correct ranking of all six study types (6/6) was achieved by four students (17.4%) in the publication group and by 13 students (4.3%) in the group without publication experience. This difference did not reach statistical significance (Fisher’s exact test, p = 0.092). Comparisons between students with a prior university degree (n = 23, 7.0%) and those without such a degree (n = 306, 93.0%) did not demonstrate a statistically significant difference (p = 0.380). Item-level analysis revealed a statistically significant difference only for the case report category (p = 0.023), with higher accuracy observed among students without a prior degree. No consistent statistically significant associations were observed between hierarchy test performance and having a physician or scientist in the family.

Sources of medicine information

The distribution of responses regarding primary sources of medical knowledge is presented in Figure 6. Academic textbooks were the most frequently reported source (n = 309, 93.9%), followed by university classes (n = 294, 89.4%). Scientific articles ranked third, being indicated by 216 respondents (65.7%). Pharmaceutical company leaflets and promotional materials (n = 16, 4.9%) and press articles (n = 34, 10.3%) were reported least frequently. Social media content was indicated by 122 respondents (37.1%), while guidelines issued by scientific institutes and medical societies were reported by 126 (38.3%).

**Figure 6:**
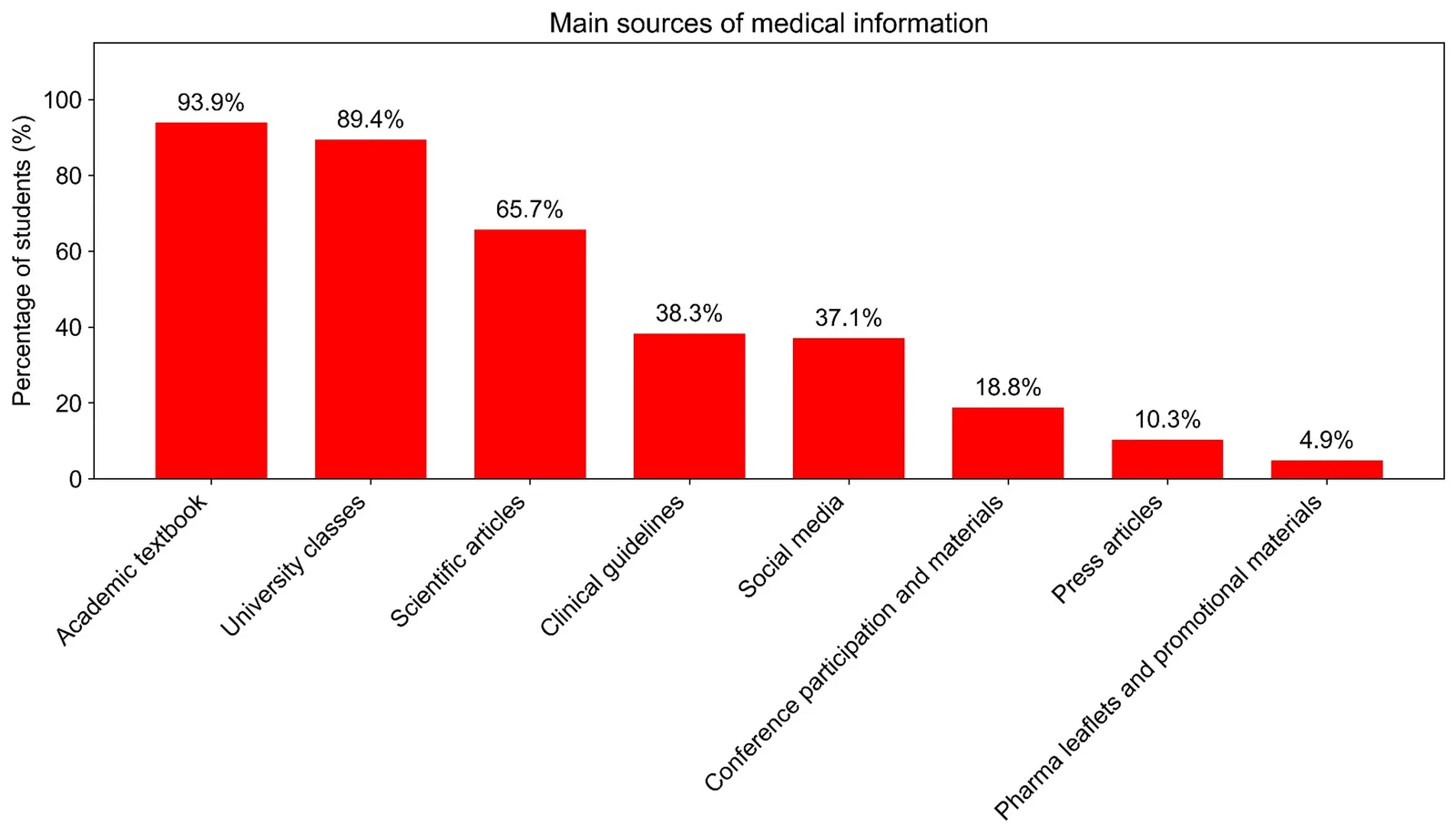
Main sources of medical information reported by medical students (multiple responses allowed)

The most frequently reported combinations of primary sources were: (1) academic textbooks, university classes, and scientific articles (50 respondents, 15.2%); (2) academic textbooks and university classes (36 respondents, 10.9%); and (3) academic textbooks, university classes, scientific articles, and guidelines from scientific societies (35 respondents, 10.6%). Across the most common combinations, academic textbooks, university classes, and scientific articles were consistently present. Among online sources of medical information, Google was the most frequently selected option (n = 271, 82.4%). Wikipedia and PubMed/Medline were each indicated by 238 respondents (72.4%), followed by the Medycyna Praktyczna portal (n = 237, 72.0%). One predefined option (bmjupdates+) did not receive any responses. Participants additionally suggested 20 other online sources, most of which were indicated by a single respondent. Among these, UpToDate (n = 25, 7.6%) and Medycyna po Dyplomie (n = 12, 3.7%) were the most frequently reported. The most common combination of online sources was Google, Wikipedia, PubMed/Medline, and Medycyna Praktyczna (46 respondents, 14.0%). This was followed by Google, Google Scholar, PubMed/Medline, Wikipedia, and Medycyna Praktyczna (26 respondents, 7.9%), and by Google and Wikipedia alone (23 respondents, 7.0%). The maximum number of online sources indicated by a single respondent was eight (n = 3, 0.9%), while 13 respondents (4.0%) reported using only one online source. When primary and online sources were considered jointly, academic textbooks, university classes, and the Google search engine were the three most frequently reported sources overall. Most respondents reported using multiple sources of medical information rather than relying on a single source.

To assess differences in the use of medical information sources, a frequency analysis was performed comparing students who had completed an EBM course with those who had not (Figure 7). In both groups, the most frequently reported primary sources were academic textbooks and university classes. No statistically significant differences were observed between the groups for these two sources (p > 0.05). Statistically significant differences were identified for the use of scientific articles (p = 0.009) and clinical guidelines (p < 0.001), both of which were reported more frequently by students who had completed an EBM course. No significant differences between groups were observed for the use of social media, press articles, conferences, or pharmaceutical company materials (all p > 0.05).

**Figure 7:**
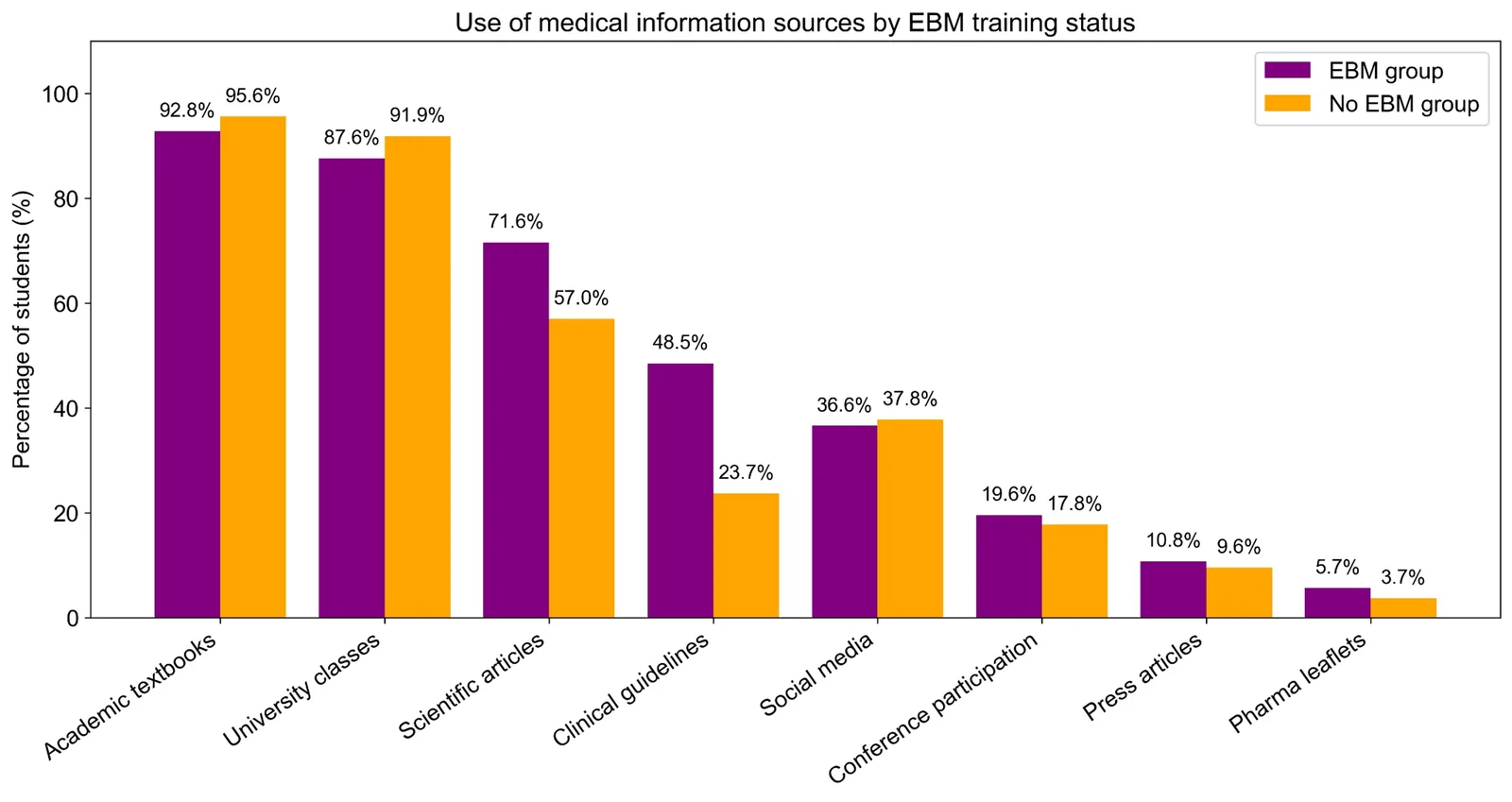
Use of main medical information sources according to participation in EBM courses Values represent percentages of students in each group (EBM group: n = 194; non-EBM group: n = 135). EBM: Evidence-Based Medicine

Table 4 presents the distribution of primary medical information sources by year of study. Across all academic years, academic textbooks and university classes remained the most frequently reported sources. Comparisons across years demonstrated a statistically significant increase in the use of clinical guidelines with advancing years of study (p < 0.001). In contrast, no statistically significant differences were observed in the frequency of using scientific articles across years of study (p = 0.186). No statistically significant decline in the use of textbooks was observed with increasing year of study (p = 0.237), despite minor fluctuations. Similarly, no significant change in the frequency of using university course materials was observed across years of study (p = 0.726).

**Table 4:** Use of main sources of medical information sources by year of study

Source	Year 1 (n = 82)	Year 2 (n = 28)	Year 3 (n = 36)	Year 4 (n = 92)	Year 5 (n = 67)	Year 6 (n = 24)
Academic textbooks	81 (98.8%)	25 (89.3%)	33 (91.7%)	87 (94.6%)	62 (92.5%)	21 (87.5%)
University classes	72 (87.8%)	26 (92.9%)	34 (94.4%)	81 (88.0%)	61 (91.0%)	20 (83.3%)
Scientific articles	47 (57.3%)	15 (53.6%)	24 (66.7%)	68 (73.9%)	45 (67.2%)	17 (70.8%)
Clinical guidelines	8 (9.8%)	4 (14.3%)	16 (44.4%)	37 (40.2%)	47 (70.1%)	14 (58.3%)
Social media	32 (39.0%)	12 (42.9%)	12 (33.3%)	34 (37.0%)	23 (34.3%)	9 (37.5%)
Conference participation/materials	11 (13.4%)	7 (25.0%)	9 (25.0%)	12 (13.0%)	17 (25.4%)	6 (25.0%)
Press articles	8 (9.8%)	4 (14.3%)	2 (5.6%)	13 (14.1%)	5 (7.5%)	2 (8.3%)
Drug leaflets and promotional materials	4 (4.9%)	1 (3.6%)	3 (8.3%)	4 (4.3%)	4 (6.0%)	0 (0.0%)

In addition to primary sources of medical knowledge, respondents were asked to indicate the online sources of medical information they most frequently used. According to the predefined categorization, internet search engines were reported by 304 students (92.4%), followed by medical portals (251 students, 76.3%), scientific databases (240 students, 72.9%), and scientific portals (239 students, 72.6%). The “Other” category comprised sources that could not be clearly classified and included five individual responses, each reported by a single participant. Among the 194 students who had completed an EBM course (59.0% of the study population), the most frequently selected categories of online sources were internet search engines (n = 176; 90.7%), medical portals (n = 173; 89.2%), and scientific databases (n = 172; 88.7%). Scientific portals were reported by 140 students (72.2%), while the “Other” category was reported by two students (1.0%). Among individual online sources used by students who had completed EBM training, the most frequently selected were PubMed/Medline (n = 171, 88.1%), Medycyna Praktyczna (n = 171, 88.1%), Google (n = 154, 79.4%), Wikipedia (n = 138, 71.1%), and Google Scholar (n = 71, 36.6%).

Database use was reported by 172 (88.7%) of students with EBM training and by 68 (50.4%) students without such training (p < 0.001). Differences were also observed for medical portals (173, 89.2% vs 78, 57.8%). Internet search engines were commonly used in both groups (176, 90.7% and 128, 94.8%, respectively). Associations between the use of specific categories of online sources and performance on the theoretical knowledge test were also examined. Use of scientific databases was positively correlated with test scores (rho = 0.249; p < 0.001) and negatively correlated with the number of “I do not know” responses (rho = -0.228; p < 0.001). Use of medical portals was also positively correlated with test scores, although the association was weaker (rho = 0.201; p < 0.001). No statistically significant association with test scores was observed for the use of internet search engines or scientific portals (p > 0.05). Use of scientific portals was weakly associated with a higher number of “I do not know” responses (rho = 0.113; p = 0.042). The relationship between the number of different online sources reported by respondents and their theoretical knowledge test scores was also assessed. A statistically significant positive correlation was observed (rho = 0.268; p < 0.001).

The frequency of use of medical portals (e.g., Medycyna Praktyczna, Medscape, Medycyna po Dyplomie) and scientific databases (e.g., PubMed, ResearchGate, Embase) was compared between younger students (years 1-3) and older students (years 4-6). Among younger students, an identical proportion reported using medical portals and scientific databases (n = 77, 52.7%) for both categories. In contrast, among older students, medical portals were reported by 172 respondents (94.0%), and scientific databases by 163 (89.1%). Differences between younger and older students in the use of both medical portals and scientific databases were statistically significant (p < 0.001 for both). The number of young students using only databases was equal to the number using only medical portals (n = 25 in each case).

An additional analysis examined the association between the use of scientific databases and authorship of scientific publications. Among students who reported using databases, 21 (8.8%) declared authorship or co-authorship of a scientific article. Among students who did not report using databases, two (2.3%) declared such authorship. This difference did not reach statistical significance (p = 0.07).

## Discussion

This study provides a comprehensive overview of medical students’ knowledge of EBM. Several consistent patterns emerge from the results and merit discussion. The results of the theoretical knowledge test reveal that the majority of participants possessed basic to moderate theoretical knowledge (concentration of results in the middle range). This distribution also suggests that the test was appropriately balanced in terms of difficulty and capable of differentiating between varying levels of competence.

The wide range in the number of “I don’t know” responses indicates marked heterogeneity in students’ confidence and awareness of their own knowledge limitations. From an educational perspective, this finding is particularly relevant, as the ability to recognize when one’s own knowledge is insufficient to answer a clinical question is a core component of EBM. In this context, “I don’t know” responses may serve as a proxy not only for knowledge gaps but also for metacognitive awareness, which is essential for safe and reflective clinical practice.

Across nearly all analyses, participation in formal EBM courses was associated with better outcomes. Students who reported having completed EBM classes achieved higher scores on the theoretical knowledge test, declared higher levels of confidence in both theoretical and practical EBM-related skills, and demonstrated greater accuracy in ranking study designs according to the hierarchy of evidence. These findings are consistent with previous reports suggesting that structured exposure to EBM principles improves both knowledge and analytical skills among medical students [2-4]. In studies where students were not exposed to structured EBM courses, knowledge and confidence in applying EBM were often limited, despite recognition of its relevance [3,4].

However, the literature is not consistent, as some studies have reported no significant relationship between prior EBM training and knowledge, attitude, or practice scores [6]. A notable finding is the strong agreement between students’ self-assessment of theoretical knowledge and their performance on the objective knowledge test. Students who declared greater familiarity with EBM and statistical terminology tended to achieve higher test scores, indicating that self-assessment, at least at the group level, reasonably reflected actual knowledge. This alignment contrasts with concerns frequently raised in the literature regarding overconfidence or miscalibration in self-reported competencies ​[12,13,16].

Previous studies have reported that self-perceived competence in EBM may correlate poorly with objectively measured skills, particularly for applied methodological competencies, while overconfidence effects have also been observed in medical student assessments, especially among lower-performing learners ​[12,17].

In contrast, self-assessment of practical skills - particularly the ability to identify areas where the literature does not allow a reliable clinical conclusion - was less strongly associated with objective knowledge. This discrepancy may reflect the inherently more abstract and experience-dependent nature of such competencies, which may develop later in medical education and require repeated exposure to real clinical uncertainty.

These findings align with research indicating that self-assessment accuracy depends on task complexity, learner experience, and the availability of feedback, with both overconfidence and underconfidence coexisting across domains and levels of difficulty ​[16-18]. Cognitive research further suggests that self-assessment is constrained by the incomplete information individuals have about their own performance, making external feedback and objective assessment essential for accurate self-evaluation, while confidence-competence alignment improves with clinical exposure and structured feedback [18,19].

Combined with evidence that self-assessment is often biased [20], these results highlight the need for structured feedback and objective assessment in EBM education to improve self-evaluation and clinical reasoning.

The hierarchy test revealed a striking pattern. Extreme categories of evidence - case reports and meta-analyses of randomized controlled trials - were relatively well recognized, whereas intermediate study designs, particularly systematic reviews of observational studies and single randomized controlled trials, posed substantial difficulties. This suggests that students may acquire a simplified, binary understanding of evidence quality (“low” versus “high”), without fully appreciating the nuances of study design and methodological rigor. Although students who completed EBM courses generally performed better in this task, complete mastery of the hierarchy remained uncommon even in this group. The reduced total classification error among EBM-trained students indicates a more structured understanding of evidence ranking, yet the overall difficulty of this task highlights an area where current educational approaches may require refinement. These results support the observation from previous studies that, although EBM training improves knowledge and skills, achieving comprehensive competence remains challenging [2,9,12,13].

Advancing year of study was consistently associated with better theoretical knowledge and greater confidence, as well as fewer “I do not know” responses. This likely reflects cumulative exposure to medical literature, clinical reasoning, and academic expectations over time. These observations are consistent with previous studies showing that more advanced students demonstrate higher EBM knowledge and confidence [2,3].

Students who reported authorship or co-authorship of scientific publications generally demonstrated higher theoretical knowledge, higher self-assessment scores, and better performance in selected components of the hierarchy test. This finding supports the notion that active engagement in research may reinforce EBM competencies [2,3]. At the same time, the lack of consistent statistical significance across all outcomes indicates that publication experience alone does not guarantee comprehensive mastery of EBM principles.

The analysis of information sources revealed a complex and realistic picture of students’ information-seeking behavior. Academic textbooks and university classes remained the most frequently reported primary sources across all years of study, and their use did not decline substantially with academic progression. Rather than being replaced, these traditional sources appeared to coexist with an expanding range of online resources. Use of scientific articles and clinical guidelines was more frequent among students who had completed EBM courses and among those in higher years of study. This pattern suggests a gradual shift toward more specialized and practice-oriented sources as students gain experience. At the same time, general-purpose search engines and widely accessible online platforms remained highly prevalent across all groups, reflecting their practical convenience in everyday information retrieval. Notably, students who reported using scientific databases tended to achieve higher scores on the theoretical knowledge test and selected the “I do not know” option less frequently. While this association does not imply causation, it suggests that engagement with structured academic resources may be linked to greater confidence and factual knowledge. Conversely, reliance on a single or limited set of online sources was associated with lower test performance, whereas using a broader range of online resources correlated with higher knowledge scores.

These results are supported by previous research. Several studies reported that traditional sources such as textbooks and lectures remain widely used across all years of study, while more advanced students increasingly access scientific articles, clinical guidelines, and databases ​[2,3,5]. Engagement with these structured academic resources has been associated with higher theoretical knowledge and greater confidence in EBM among students.

Study limitations

Several limitations of this study should be acknowledged. Although efforts were made to recruit a large and diverse sample of medical students, participation rates across universities did not reflect the actual distribution of medical students in Poland. Despite repeated invitations and reminders, some universities were underrepresented, which may have resulted in selection bias. Students who are academically active or engaged in university-related activities may therefore be overrepresented in the study sample. In addition, the distribution of respondents across years of study was uneven, with fourth-year students constituting the largest proportion of participants and sixth-year students being relatively underrepresented. This imbalance may have influenced comparisons across academic years. Participation in EBM courses was assessed using a binary self-report item (“participated” vs. “did not participate”). No information was collected regarding the duration, intensity, content, or quality of these courses, nor about students’ attendance or subjective evaluation of the teaching. The questionnaire did not collect demographic data such as age, gender, or place of residence. As a result, potential associations between demographic characteristics and EBM knowledge or competencies could not be examined. Some subgroups included in the analyses, particularly students with prior university degrees and those reporting authorship of scientific publications, were relatively small. This may have increased variability and reduced the statistical power to detect differences, and the corresponding results should therefore be interpreted with caution. Although nonparametric statistical methods were applied, which are considered robust to unequal group sizes, the generalizability of these findings remains limited. The study was conducted only among medical students at Polish medical universities, which may limit the applicability of the study to other countries and medical education systems. Data collection was conducted in two separate periods (2020 and 2025), which may have introduced heterogeneity related to changes in medical education, increased availability of digital resources, and the broader impact of the COVID-19 pandemic. These temporal factors were not explicitly controlled for in the analyses. Despite these limitations, the study provides a comprehensive overview of EBM knowledge, self-assessed competencies, and information-seeking behaviors among medical students and highlights important areas for further research and educational development.

## Conclusions

Taken together, these findings point to several implications for medical education. First, formal EBM instruction appears to be associated with measurable benefits across multiple domains, supporting its inclusion as a core component of the medical curriculum. Second, difficulties in understanding intermediate levels of evidence suggest that teaching should place greater emphasis on study design nuances rather than solely on the extremes of the evidence hierarchy. Third, fostering active engagement with scientific literature and research activities may further enhance students’ EBM competencies. At the same time, the persistence of traditional learning resources alongside digital tools underscores the importance of integrating, rather than replacing, established educational methods with newer approaches. Helping students critically navigate the growing diversity of information sources remains a key challenge for contemporary medical education.

## Appendices

Appendix A

**Table 5:** The baseline characteristics of medical students - participants of the questionnaire (N = 329)

Variable	Medical students, N = 329
University	
Collegium Medicum of University of Warmia and Mazury in Olsztyn	160 (48.6%)
Collegium Medicum of Jagiellonian University in Krakow	40 (12.2%)
Medical University of Lublin	35 (10.6%)
Collegium Medicum of University of Rzeszow	20 (6.1%)
Collegium Medicum of Jan Kochanowski University in Kielce	14 (4.3%)
University of Opole	14 (4.3%)
Medical University of Bialystok	6 (1.8%)
Collegium Medicum in Bydgoszcz of Nicolaus Copernicus University in Torun	6 (1.8%)
Medical University of Silesia in Katowice	6 (1.8%)
Wroclaw Medical University	6 (1.8%)
Poznan University of Medical Sciences	5 (1.5%)
Other	5 (1.5%)
Pomeranian Medical University in Szczecin	4 (1.2%)
Medical University of Warsaw	4 (1.2%)
Collegium Medicum, University of Zielona Góra	2 (0.6%)
Andrzej Frycz Modrzewski Krakow University	1 (0.3%)
Medical University of Lodz	1 (0.3%)
Medical University of Gdansk	0 (0%)
Cardinal Stefan Wyszynski University	0 (0%)
Lazarski University in Warsaw	0 (0%)
Academy of Silesia	0 (0%)
Year of study	
1st year	82 (24.9%)
2nd year	28 (8.5%)
3rd year	36 (10.9%)
4th year	92 (28.0%)
5th year	67 (20.4%)
6th year	24 (7.3%)
Master degree (or equivalent)	
Yes	23 (7.0%)
No	306 (93.0%)
Scientific publication (author/co-author)	
Yes	23 (7.0%)
No	306 (93.0%)
Family member is a physician	
Yes	92 (28.0%)
No	237 (72.0%)
Family member is a scientist	
Yes	61 (18.5%)
No	268 (81.5%)
Participation in EBM classes	
Yes	194 (59.0%)
No	135 (41.0%)

Appendix B

**Table 6:** Questionnaire: knowledge of Evidence-Based Medicine (EBM) and statistics in medical research

Item	Question	Response options
1	Year of study	I; II; III; IV; V; VI
2	University	Medical University of Lodz
		Poznan University of Medical Sciences
		Medical University of Silesia in Katowice
		Medical University of Warsaw
		Medical University of Lublin
		Medical University of Bialystok
		Medical University of Gdansk
		Pomeranian Medical University in Szczecin
		Wroclaw Medical University
		Collegium Medicum in Bydgoszcz of Nicolaus Copernicus University in Torun
		Collegium Medicum of Jagiellonian University in Krakow
		Collegium Medicum of University of Warmia and Mazury in Olsztyn
		Collegium Medicum, University of Zielona Gora
		Collegium Medicum of Jan Kochanowski University in Kielce
		Collegium Medicum of University of Rzeszow
		Cardinal Stefan Wyszynski University
		Lazarski University in Warsaw
		University of Opole
		Andrzej Frycz Modrzewski Krakow University
		Academy of Silesia
		Other
3	Have you previously completed another degree programme and obtained at least a Bachelor's/Engineering degree?	Yes / No
4	Is anyone in your family or close relatives a physician?	Yes / No
5	Is anyone in your family or close relatives a scientist?	Yes / No
6	Does your study programme include classes on EBM?	Yes / No / I don't know
7	During your studies so far, have you had classes on EBM?	Yes / No
8	If yes, what grade did you receive?	2.0; 3.0; 3.5; 4.0; 4.5; 5.0; Pass without grade
9	Have you been an author/co-author of a paper (other than a case report) published in a peer-reviewed journal?	Yes / No
10	If yes, in which journal?	Open-ended response
11	What is your main source of medical information? (multiple answers allowed)	Academic textbooks
		University classes
		Scientific articles
		Press articles
		Social media
		Clinical guidelines
		Leaflets and promotional materials from pharmaceutical companies, etc.
		Conference participation and materials
12	Which online sources do you use when searching for medical information? (multiple answers allowed)	Google
		Google Scholar
		Wikipedia
		PubMed/MEDLINE
		Medscape
		Cochrane Library
		Scopus
		Web of Science
		Embase
		Ovid
		bmjupdates+
		NICE
		Medycyna Praktyczna
		UpToDate
		Other
13	Do you know what the following terms mean?	
13.1	Original article	Yes / No
13.2	Review article	Yes / No
13.3	Case report	Yes / No
13.4	Cohort study	Yes / No
13.5	Prospective study	Yes / No
13.6	Retrospective study	Yes / No
13.7	Cross-sectional study	Yes / No
13.8	Case-control study	Yes / No
13.9	Phase I clinical trial	Yes / No
13.10	Phase II clinical trial	Yes / No
13.11	Phase III clinical trial	Yes / No
13.12	Medical experiment	Yes / No
13.13	Meta-analysis	Yes / No
13.14	Systematic review	Yes / No
13.15	p-value	Yes / No
13.16	Confidence interval	Yes / No
13.17	Median	Yes / No
13.18	Mean	Yes / No
13.19	Mode	Yes / No
13.20	Standard deviation	Yes / No
13.21	Type I error	Yes / No
13.22	Type II error	Yes / No
13.23	Odds ratio	Yes / No
13.24	Specificity	Yes / No
13.25	Sensitivity	Yes / No
13.26	Relative risk	Yes / No
13.27	Randomization	Yes / No
13.28	Evidence-based medicine	Yes / No
14	Rate the following skills on a scale from 1 to 5	
14.1	Critical appraisal of scientific literature	1; 2; 3; 4; 5
14.2	Identification of areas where there is insufficient high-quality evidence to answer a clinical question	1; 2; 3; 4; 5
15	A cross-sectional study is:	
	A study in which the occurrence of a specific endpoint is assessed in exposed and unexposed groups of individuals who were free of the endpoint at baseline	
	A study examining the relationship between the occurrence of a specific disease and exposure to a given factor in a defined population at a single point in time	
	A study in which patients are randomly assigned to an experimental group receiving the intervention or to a control group, and are then observed for the occurrence of specific endpoints	
	I do not know	
16	In a case-control study:	
	Patients with a given clinical condition are selected first, and then each of them is matched with one or more patients without that condition	
	The occurrence of a specific endpoint is assessed in groups of individuals exposed and unexposed to a given factor	
	The prevalence is assessed, that is the number of individuals with a specific clinical condition at a given point in time	
	I do not know	
17	The mode is:	
	The value occurring most frequently in a given distribution	
	The number of cases occurring most frequently in a given study	
	The difference between the most frequent (dominant) values and other values	
	I do not know	
18	Standard deviation is:	
	A value describing the average deviation from the arithmetic mean	
	A value describing the average deviation from the median	
	A value describing deviation of the median from the mean	
	I do not know	
19	Type I error is:	
	Rejecting the null hypothesis assuming it is true	
	Accepting the null hypothesis assuming it is false	
	Rejecting the alternative hypothesis assuming it is true	
	I do not know	
20	A confidence interval is:	
	A range of values likely to contain the true population parameter. The narrower it is, the more precise the estimate	
	A range of values within which, if the obtained result falls, it should by definition be considered incorrect	
	Another term for statistical significance, represented by the index “95% CI”	
	I do not know	
21	Phase II clinical trial:	
	It involves assessing the safety and dosage of the investigational drug. It is conducted in a small group of healthy volunteers	
	It involves assessing the safety and efficacy of the investigational drug. It is conducted in a relatively small group of patients	
	It involves laboratory and pharmaceutical research aimed at identifying a new molecule that, in theoretical models, could treat a given disease	
	I do not know	
22	Relative risk is:	
	The ratio of the probability of a specific clinical outcome occurring in the group exposed to a given factor to the probability of that outcome occurring in the control group	
	Number of patients who need to be treated with a given intervention over a specified period of time in order to prevent one adverse endpoint	
	The ratio of the probability of occurrence of an endpoint in the experimental group receiving the evaluated intervention to the probability of that occurrence in the control group	
	I do not know	
23	Randomization is:	
	Qualitative synthesis of results of studies addressing the same clinical question	
	The process of randomly assigning study participants to an experimental group and a control group	
	It determines the level of precision of a given estimate. The greater the randomization in a study, the greater the statistical significance of the result	
	I do not know	
24	EBM means:	
	Medicine based on facts/scientific evidence	
	A contemporary trend in medicine requiring physicians to make decisions based only on the latest scientific evidence	
	Basing medicine on the results of one’s own published scientific research	
	I do not know	
25	Rank the following study types by increasing hierarchy of evidence (1-6, where 6 is the most reliable):	
	Systematic review of observational studies	
	Meta‑analysis of randomized controlled trials	
	Case report	
	Single randomized controlled trial	
	Single observational controlled study	
	Nonrandomized controlled experimental study	

Appendix C

**Table 7:** Cohen's kappa coefficient describing the results for validation of the repeatability of responses Statistical significance: * p<0.05 , ** p<0.01, *** p<0.001 The values of the coefficients were tested with Cohen's kappa significance test used to verify the hypothesis that kappa = 0, indicating the randomness of the results of double measurements of responses. Cohen's kappa coefficient describes the degree of agreement of duplicate measurements of the same variable; it is determined for dependent categorical variables, and its value ranges from -1 to 1, where the value 1 means full compliance, and the value 0 means compliance at a random level. In this study, to interpret the results of the statistical analysis of repeatability, a conventional scale was used, assigning the following meaning to individual values of Cohen's kappa coefficient: 0.81-1.00 very good repeatability, 0.61-0.80 good repeatability, 0.41-0.60 moderate repeatability, 0.20-0.40 poor repeatability, <0.21 very poor repeatability.

Question	Cohen's kappa	Question	Cohen's kappa
13.1	0.872***	13.21	0.573***
13.2	1.000***	13.22	0.826***
13.3	0.789***	13.23	0.826***
13.4	0.789***	13.24	0.826***
13.5	0.671***	13.25	0.729***
13.6	0.685***	13.26	1.000***
13.7	0.568***	13.27	0.855***
13.8	0.627***	13.28	1.000***
13.9	0.627***	14.1	0.694***
13.10	0.627***	14.2	0.850***
13.11	0.826***	15	0.629***
13.12	0.791***	16	0.816***
13.13	0.659***	17	1.000***
13.14	0.774***	18	0.841***
13.15	0.671***	19	0.762***
13.16	1.000***	20	0.782***
13.17	1.000***	21	0.835***
13.18	0.826***	22	0.683***
13.19	0.826***	23	0.657***
13.20	0.573***	24	1.000***
